# Accessing seasonal weather forecasts and drought prediction information for rural households in Chirumhanzu district, Zimbabwe

**DOI:** 10.4102/jamba.v11i1.777

**Published:** 2019-10-07

**Authors:** Mashoko S. Grey

**Affiliations:** 1 CSR Group Africa Consultancy, Harare, Zimbabwe

**Keywords:** drought prediction, seasonal weather forecasts, indigenous knowledge, livelihoods, early warning system, vulnerability, hazard

## Abstract

Seasonal weather forecasts and drought hazard prediction through media sources and indigenous knowledge help provide an understanding of early warning systems and the preferred source information by rural households. This article focuses on the investigation of households’ access to weather forecasts and drought hazard prediction information as early warning to reduce drought risk on livelihood activities. The study was carried out in Chirumhanzu district, and the methods used for data collection included 217 household surveys, six focus group discussions, key informants’ interviews and document review. The study found that the majority of the households in the study area had access to seasonal weather forecast information (scientific), which almost half of the respondents received through radios. However, vulnerability to climate risks was exacerbated by seasonal weather forecasts, which were deemed by some households to be unreliable, inaccurate and not easily understood. In this regard, some households used indigenous knowledge to inform them on the status of the incoming rainy season and drought prediction. The use of indigenous knowledge depended on individuals’ ability to read and decode natural indicators of seasonal weather forecast and drought prediction. Indigenous knowledge is valuable for climate science as it enhances observations and interpretations on a larger spatial scale with considerable temporal depth by highlighting elements that are measured by climate science. Both scientific weather information and indigenous knowledge are important for seasonal weather forecasting and drought prediction, especially in rural settings, and complement each other if used and availed timely to households.

## Introduction

The purpose of this article is to critically understand how households and communities access seasonal weather and drought prediction information as this shapes strategies for protecting their livelihood activities. By and large, these perceptions are moulded by the availability of and access to weather information through different sources of conventional and/or through understanding indigenous knowledge systems. An appreciation of these sources of seasonal weather forecasts and drought prediction, including conventional media sources and indigenous knowledge, helps provide an understanding of seasonal weather dynamics and early warning systems (EWS) for hazards like drought in the community and, more importantly, the source of weather information preferred by rural households.

Studies in Zimbabwe indicate that smallholder farmers are increasingly concerned about unfamiliar climate dynamics, including uncertainty about planting, loss of crops and damage to infrastructure (Chirau, Nkambule & Mupambwa 2014; Jiri et al. 2016; Zvigadza, Mharadze & Ngena 2010). This is largely because the meteorological rainfall forecasts available through various channels are not readily accessible to rural communities (Chisadza et al. 2013). Furthermore, rural smallholder farmers in Zimbabwe highlight the lack of access to seasonal weather trends and climate information (Nangombe 2013). The EWS for drought in Zimbabwe at the national level is not very effective leaving smallholders to engage in their livelihood activities without adequate information for the rain season and more so for potential drought hazards (Chisadza et al. 2013). Yet, most communal areas in Zimbabwe rely much on natural rains such that accuracy in weather prediction results would improve farmers yield and productivity (Risiro et al. 2012).

Knowledge about weather forecasts and drought hazard prediction might depend on, among other factors, access to sources of weather information and the interest of individuals to know and learn about local seasonal weather trends over the years. However, droughts are generally classified into four types: meteorological, hydrological, agricultural and socio-economical drought. This article is focused on meteorological drought. Meteorological drought is defined as lack of rainfall for a long period over an area (Wilhite 2000, 2005). Informed households are presented with a greater opportunity to make informed farming decisions and implement the necessary risk reduction and coping strategies (Peters 2015). In this article, an EWS is defined as a series of organised surveillance mechanisms or actions that collect information on potential hazards in a given location in order to trigger timely, coordinated responses (FAO 2014). Wealthy households with access to electronic and/or print media have better access to seasonal weather forecasts and drought predictions than poorer households (Peters 2015). As a result of low wealth levels in rural areas, most households have no access to seasonal weather information and therefore limited capacity to implement climate change adaptation (CCA) and drought disaster risk reduction (DRR) strategies. These households consequently suffer from the adverse impacts of various climate risks including droughts (Chirau et al. 2014; Doldman & Mitlin 2015; IPCC 2014; Peters 2015).

Even with full access to seasonal weather forecasts and drought predictions, there will always be shortcomings on the part of households to translate weather information into risk reduction and adaptation action (Nhemachena et al. 2014). Taking appropriate action requires availability of risk reduction information, accurate information, understanding of this information, and more importantly the capacity to implement desired strategies (Dejene et al. 2011; Heltberg, Siegel & Jorgensen 2015; Noble et al. 2014). Disseminated weather information is therefore vital for the rural households to be adequately prepared to protect their livelihood strategies in the event of a hazardous episode, but this weather information also needs to come with other knowledge and forms of support (Peters 2015). The timing and form of weather information, access to trusted guidance, and ability to interpret and implement the information in decision-making processes, are important to rural households for building resilient livelihoods (Wilhite, Sivakumar & Pulwarty 2014). Ziervogel, Garderen and Price (2016) maintain that it is important to have access to usable weather information that is considered alongside the socio-economic and governance context. In addition to the above, Gautier, Denis and Locatelli (2016) argue that there is a need to take local knowledge and information into account when dealing with climate hazards, as its abandonment may increase the vulnerability of local households and communities. Therefore, in this context, failure to access, understand, and/or translate weather information (local or scientific) into appropriate action by poor households might result in adverse drought impacts (Gautier et al. 2016; Heltberg et al. 2015; Peters 2015).

Seasonal weather and drought prediction information received by households shapes their perceptions of climate variability and change and associated risks (Janicot et al. 2015). Bryan et al. (2013) similarly argue that smallholder farmers’ behaviour is shaped more by their perceptions of climate variability and climate risk than by actual weather patterns as measured by scientific methods. They further maintain that while farmers’ perceptions are based in part on past observations, several studies have suggested that farmers place greater emphasis on recent weather events (weather forecasts and drought prediction) in forming their perceptions of climate risk and in making decisions about risk reduction and adaptive strategies. Perceptions of weather signals and the ability to anticipate a change in the season is thus a determinant stage in the management of a farming system for smallholder farmers (Janicot et al. 2015; Simba, Chikodzi & Murwendo 2012). While smallholder farmers and rural households may perceive changes in weather patterns, these will be taking them into unfamiliar experiences and situations, and therefore weather information needs to be accessed by all households.

## Methodological approach

### Study area

**FIGURE 1:**
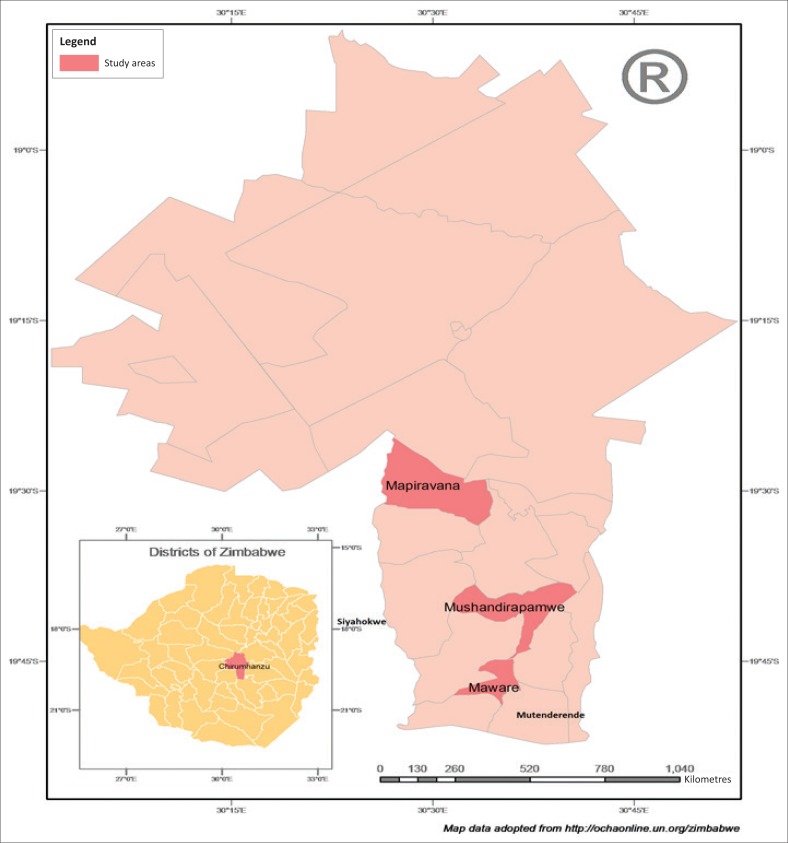
Map of the study area.

The location of the study was Chirumhanzu rural district (Midlands province) in Zimbabwe as shown in Figure 1. The Midlands province is further divided into eight districts. Chirumhanzu district is divided into 25 administrative wards (Figure 1). Chirumhanzu district has a total of 19 736 households and a total population of 81 087 with 47% males and 53% females (ZimStat 2012). Chirumhanzu district lies in agro-ecological regions 3 and 4 where semi-intensive mixed farming and extensive farming with livestock ranching are suitable and recommended. The district is located mainly in the mid-altitude areas of the country and is characterised by an annual rainfall of 500 mm – 750 mm, mid-season dry spells and high temperatures (Figure 1; FAO 2006; Mugandani et al. 2012). The district was therefore purposively selected, as it is one of the areas frequently affected by hydro-meteorological disasters, specifically droughts, over the years.

## Methods

This study used data collection methods that collect both qualitative and quantitative data. The use of this approach was premised on the basis that all methods have inherent biases and strengths; using a mixed methods approach spreads the prospect that data collected will be of greater quality (Creswell & Plano Clark 2007; Denscombe 2008; Johnson, Onwuegbuzie & Turner 2007). This integration of approaches generated deeper and broader insights and a full understanding of the complex social processes required for DRR, particularly droughts and CCA in relation to early through access to climate information (Johnson et al. 2007). Further, this approach broadened the sample size, as some household heads not included in the survey administration, were likely involved in focus group discussions (FGDs) and participatory learning and action methods (PLAMs) (Denscombe 2008).

Household survey questionnaires were administered to 217 respondents^1^ across three randomly selected wards. The sample size selection for the households assumed that 25% of the rural population has been affected by drought, with a desired 95% confidence interval and precision of 0.05% as indicated in the formula below:

$$n=t^{2}\text{x }p(1-p)=1.645^{2}\times 0.25(1-0.25)\;m^{2}0.05^{2}=203$$ [Eqn 1]

Where *n* = sample size; *t* = confidence level at 95% level of significance (1.96).

Based on the above calculation, the resultant sample size for the total households interviewed for the three wards was therefore: 203*1.5*0.05 = 218 households.

Data were collected through document review, key informant interviews (KIIs), household surveys, FGDs, seasonal calendars and transect walks. Documents reviewed included reports from the Ministries of Environment, Local Government and Agriculture, rainfall and temperature data from Meteorological Services Department (MSD), census reports and the Zimbabwe Vulnerability and Assessment Committee (ZimVAC) reports.

The household questionnaire focused on access to climate information through conventional and indigenous sources (EWS) with specific detailed aspects on each theme. A total of six FGDs (FGDs – two FGDs per ward with one for men and one for women) were held to elicit ideas, insights and experiences in a social context, where people were stimulated to give their own views (Mubaya & Yanda 2010). FGDs were held separately to encourage women to respond freely as men usually dominate discussions in this rural setting. Ten KIIs in government departments and development partners were conducted at district and national levels mainly to get policy views and technical information not attainable at the household level. Transect walks provided an opportunity to capture the current agricultural practices and other livelihood strategies for different households and communities.

## Data analysis

As the study collected both qualitative and quantitative data, the data analysis methods used matched the nature of the data as indicated below. Qualitative data were collected as interview scripts and observational notes from key informant interviews, transect walks and FGDs. The interview and discussion recordings were transcribed for analysis. For this study, thematic qualitative data analysis was employed in the rigorous ordering and structuring of the qualitative data. Thematic data analysis is the method of ‘identifying, analysing, and reporting patterns (themes) within data’ and it is also a descriptive method that reduces the data in a flexible way that dovetails with other data analysis methods (Castleberry & Nolen 2018:808). The themes were then linked together into chains or patterns of evidence to enable the drawing up of contrasts and comparisons in the experiences of the community. The Social Package for Social Sciences (SPSS) Software Version 20 was used to analyse quantitative data collected from household surveys. Responses to the household survey questions were coded for entry into the SPSS software after the data collection process. The SPSS file was used to generate frequency tables, percentages of responses and cross-tabulations. Overall, the analysed data were presented using tables, graphs and charts.

### Ethical considerations

The permission to collect data was given by Provincial Administrator for Midlands province and the District Administrator for Chirumhanzu district.

## Results and discussion

### Conventional sources (early warning system) for seasonal weather forecasts and drought hazard prediction

**FIGURE 2:**
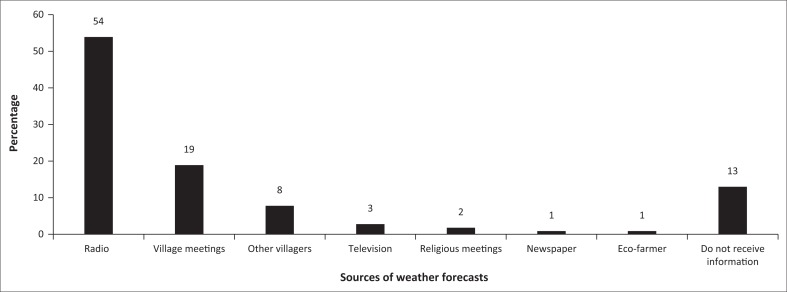
Conventional sources of weather forecasts (*n* = 217).

It is important to understand the role of access to seasonal weather forecasts and drought prediction information in helping households and smallholder farmers to make decisions regarding their livelihood activities. Almost half (54%) of the respondents mentioned radios, 19% stated village meetings and 8% mentioned other villagers as sources of information (Figure 2). The finding that almost half of the respondents received weather information through the radio is aligned to household asset ownership. Regarding the latter, this would have been from villagers who attended the village/ward meetings or those with access to media sources. As only 3% owned television sets, others mentioned getting the information from religious meetings (2%), newspapers (1%) and EcoFarmer facility (1%), while 13% did not have access to seasonal weather forecasts and drought prediction (Figure 2).

Although the majority of respondents (87%) had access to seasonal weather forecasting and drought prediction through various sources, this did not automatically translate into adaptation or risk reduction actions. Respondents mentioned several factors that influenced whether households took risk reduction action or not. These factors included the understanding of weather forecasts, perceived accuracy of the information, timeous receipt of the weather information, and the ability to translate the information into farming decisions.

Fifty-four per cent of respondents who received seasonal weather forecasts did not understand it, while 46% understood the weather forecasts. In support of the fact that some households often struggled to understand seasonal weather forecasts, an MSD representative revealed:

‘… the major challenge is understanding of the terms we use when disseminating weather information. But, in some places where we have had workshops there is now better understanding of our risk warnings and weather forecasts. For example, in Zvishavane, Gutu and Chirumhanzu districts where we had workshops there is a very good understanding of these terms and seasonal weather forecasts in general *…’* (KII with Climate Scientist from MSD, male)

Further evidence from the MSD representative indicated that in-as-much as they wanted to go to grassroots level and simplify the seasonal weather forecasts for improved understanding by rural households, resources required for such activities are immense and might not be sustainable in the long run for the department. Therefore, MSD continued disseminating the seasonal weather forecasts through Agritex, which already has structures and personnel in place at the grassroots level across the country. However, Agritex officers are not able to fully understand weather data trends and simplify it for smallholder farmers’ consumption, nor to make weather projections meaningful for farming activities considering that they do not have training on climate variability and change, climate modelling or risk reduction.

Secondly, household perceptions of the accuracy of seasonal weather forecast and drought prediction information played an important role on whether households took action or not. Households that received weather information through media sources were asked for their perception of accuracy and reliability of the information. Forty per cent of respondents believed weather information to be fair, while 24% believed it was accurate. Thirty-six per cent of respondents perceived seasonal weather forecasts as inaccurate. In support to inaccuracy, the MSD representative stated that:

‘… we are prepared in dealing with these hazards but the main problem is the MSD forecasts are not 100% accurate. They are usually 50% – 75% accurate. We also experience changes in phenomena and we have no updates, for example we had a hailstorm 2 years ago in Zvishavane and we never had a forecast on that …’ (KII with Civil Protection Unit Personnel, female)

Seasonal weather information provided by MSD has a huge geographical coverage and is not pinned down to specific area locations. Therefore, they do not have high-resolution data needed to improve the accuracy of the information disseminated. The MSD representative further supported this view and mentioned:

‘… specification of the weather forecast is quite a concern. We would like to pinpoint areas but your time allocation on television and radio is also restrictive. We would otherwise have 2 min to cover 57 districts, which is difficult. So people do not trust the weather forecast because they do not understand the trends. The small projects that we have worked with so far like International Crops Research Institute for the Semi-Arid Tropics (ICRISAT), successes were recorded when forecasts were sent via SMS every 10 days … When we go into the season, the 10-day forecasts are provided, we have the models, which we tend to generalize. They cannot be specific. If you want to try and downscale the models to some small areas, it is quite difficult *…’* (KII with Climate Scientist from MSD, male)

Thirdly, risk reduction action or non-action was attributed to timeous receipt of seasonal weather forecasts and drought prediction information. An Agritex officer from the Mushandirapamwe ward pointed out, ‘… the timely dissemination of weather information to the farmers is likely to equip them with a decision-making tool for the new cropping season …’. Forty-four per cent of respondents received seasonal weather forecast information less than 1 month before the start of the rainy season, while 27% received seasonal weather predictions 1–2 months before the start of the planting season. Lastly, 29% of respondents received information 3 months or more prior to the cropping season. The MSD representative indicated that they started issuing seasonal forecasts for the upcoming cropping season in August, which were sent through to Agritex officers who then disseminated the information to smallholder farmers around October or November in their respective areas. One Agritex officer indicated that there was often a delay in the final dissemination of seasonal weather forecasts to smallholder farmers, and this was partly attributed to bureaucratic processes and limited coordination on information flow between inter- and intra-ministry departments.

Despite high ownership of mobile phones, there was very little usage of the EcoFarmer facility (1%). This limited utilisation of the EcoFarmer facility to receive weather information can be attributed to the cost factor to the users ($1.50 per month or $0.08 per day to receive messages only without the insurance aspect) and the lack of high-quality resolution (too general) for smallholder farmers to understand and take any meaningful action. Therefore, households indicated that they were not in a position to pay daily or monthly subscriptions for services from the EcoFarmer facility specifically to receive weather information in the form of a short message service (SMS). However, making farming decisions without using weather forecast information exposes the farmer to climate risk and could result in crop failure or reduced productivity as farming activities might not be aligned to rainfall patterns.

The mass media through which households receive weather information (radio, televisions and newspapers) is expensive for rural households considering their high poverty levels (ZimVAC Report 2013). Evidence from the ZimVAC Report (2013) supports this view as it pegged the rural poverty rate at 76% and 23% within the poor and extremely poor categories respectively in Zimbabwe. In fact, some of the respondents who indicated that they rely on radio for weather information were reliant on radios from shopping centres and next-door neighbours. Indeed, in some parts of the Chirumhanzu district (the study site), one cannot receive a radio signal or a mobile network service, making it difficult for those in remote and marginalised communities to access vital seasonal weather forecasts and drought prediction to engage risk reduction and adaptation strategies.

There were differences in language and terminologies used for forecasts by MSD, which if simplified might result in improved understanding of weather forecast information at the grassroots level. In a similar study in Zimbabwe (Tsholotsho, Murehwa and Chiredzi districts), Soropa et al. (2015) showed that most smallholder farmers could not access, interpret and use scientific meteorological predictions because of their limited ability to translate weather information into action at the household level. It is important to disseminate weather information in a format and language that can be easily understood (Soropa et al. 2015). Gandure, Walker and Botha (2013) note that lack of access to early seasonal weather forecasts and unreliability of seasonal forecasts is a barrier to promoting risk reduction and adaptation action. Therefore, with limited access to simplified seasonal weather forecast information, rural households cannot position themselves strategically against drought hazards, and this creates a platform for disaster occurrences (Wilhite et al. 2014).

The accuracy of seasonal weather forecast is crucial in making farming decisions such as type and variety of crop to plant and when to plant. The MSDs’ limited airtime on media mentioned earlier, might suggest a compromised quality of weather forecast information, affecting the decisions on risk reduction and adaptation. In this regard, Wilhite et al. (2014) argue that the ability of a smallholder farmer to make an informed decision is dependent on the accuracy of available information and the manner in which it is processed at the household level. Seasonal weather forecasts need to be of high quality and context-specific and must deal with current and expected weather trends and their impacts (Gandure et al. 2013). Smallholder farmers who perceived seasonal weather forecasts as not accurate are more likely not to use the forecasts for planning their farming activities. Such scepticism by smallholder farmers might lead to non-action, even when facing imminent drought, exposing them to climate risk (Wilhite et al. 2014). Peters (2015) and Ziervogel (2016) note that seasonal weather forecasts and drought prediction can only be useful when accurate, simplified and timely in reaching the smallholder farmers and households. Yet, Agritex officers are often late in receiving seasonal weather forecasts from their line Ministry to disseminate to smallholder farmers, and by then some farmers would have already purchased inputs in preparation for the new cropping season. In some cases, these farming inputs were inappropriate for the forthcoming cropping season exacerbating the impacts of drought at the household level.

### Indigenous indicators for seasonal weather forecasting and drought hazard prediction

**FIGURE 3:**
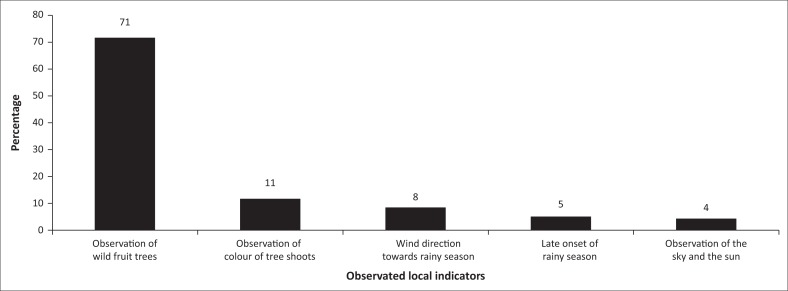
Local indicators for seasonal weather forecasting and drought prediction (*n* = 217).

Half of the respondents (53%) confirmed using indigenous knowledge^2^ against 47% who did not. These households used indigenous knowledge based on local indicators from the natural ecosystem to predict the outlook of the new cropping season or drought hazards before its commencement. The most common local indicator for drought prediction was the bearing of large quantities of wild fruits by specific trees, especially *Parinari curatellifolia, Lannea discolor* and *Lannea edulis* (71%), while observing the skies and the sun (4%) was the least-mentioned indicator (Figure 3). Other indigenous indicators used by households included observation of colour of new shoots (11%), wind direction towards the rainy season (8%), and late onset of the rains (5%) (Figure 3). Following up on bearing of wild fruits, FGD participants in Mapiravana and Mushandirapamwe wards expressed that in each year there was drought and there was an abundance of *Parinari curatellifolia* (*chakata*) fruits. However, respondents in the Maware ward mentioned that *Lannea discolor* (*gan’acha*) fruits were more pronounced in their area during droughts. Different geographical locations had different indicators for weather forecasting and drought prediction.

Dwelling on the ‘bearing of wild fruits’ indicator, study participants held that if they see *Parinari curatellifolia* (*muchakata* or *hacha*), *Lannea discolor* (*mugan’acha*) or *Lannea edulis* (*mutsambatsi*) fruit trees bearing large quantities of fruits before the rain season, then this is a sign of little rainfall (drought). The use of these indicators for weather forecasting was not unilateral, but one could use a combination of indicators in trying to gain greater accuracy. This natural mechanism of ‘bearing large quantities of wild fruits’ helped to provide a relief to the rural households from acute food shortage and hunger during drought by providing alternative food sources.

Eleven per cent of respondents indicated that if the *Brachystegia spiciformis* (*msasa*) tree shoots were dark maroon in colour, this is an indication of imminent drought and that farmers should plant small-grain crops (finger millet, millet and sorghum) that was resembled by the colour of the new shoots. Further evidence from FGDs indicated that these shoots appear just before the onset of the rain season around September/October when smallholder farmers are making farming preparations for the oncoming cropping season. For example, FGD participants in Mapiravana ward indicated that based on the *Brachystegia spiciformis* tree shoots’ colour at the onset of the 2014–2015 rainy season, a drought was predicted by the communities, and it actually occurred, highlighting local belief in the reliability of local indigenous knowledge indicators.

Further consultations during FGDs and information from the household surveys indicated that respondents believed that when the winds blow in a south to north direction in the rainy season, this indicates little rainfall. If the winds are strong and blowing from the north to the southeast, it was a sign of good rains in that particular community. It was further revealed during the FGDs that the winds could be strong and shift the clouds that should bring good rains to their locality.

In support of the importance of the local indigenous knowledge in weather and hazard prediction, the Department of Civil Protection (DCP) representative cited:

‘… we also rely on the communities for early warnings as each community has its own way of weather forecasting using their understanding of nature. In fact, we have to improve on the documentation of the local knowledge systems as they have proved to be quite accurate over the years …’ (KII with Department of Civil Protection Personnel, female)

**FIGURE 4:**
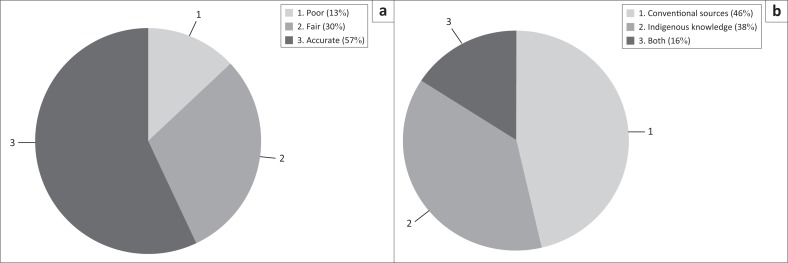
(a) Household’s perceptions of accuracy of indigenous indictors for seasonal weather forecasting and (b) preferences on sources of seasonal weather forecasting (*n* = 217).

However, the use of indigenous knowledge by local smallholder farmers in making farming decisions was largely dependent on their perception of its accuracy. However, the perception of accuracy of indigenous knowledge varied among different households, with 57% indicating that it was accurate (Figure 4). Thirty per cent of respondents mentioned that indigenous knowledge’s accuracy was fair (Figure 4). Only 13 % of respondents declared the accuracy of indigenous knowledge to be poor (Figure 4). Additionally, respondents were asked about their preferred source of seasonal weather forecasts and drought prediction. Respondents’ preferences were slightly biased towards using conventional sources (46%) (Figure 4). A smaller proportion of respondents (38%) preferred using indigenous knowledge, while 16% preferred using both (Figure 4).

Households also relied on indigenous knowledge for seasonal weather forecast and drought prediction. Households used local indigenous indicators with which they were familiar with and were able to decode. Respondents who used indigenous knowledge believed in their local indicator interpretations. Although communities are equipped with traditional knowledge and wisdom, there is a need to link indigenous knowledge and new scientific practices and policies to enable smallholder farmers to cope with climate risks, thereby providing them with the means to sustain their livelihood activities (Shaw, Pulhin & Pereira 2010). In their study in Northwest Zimbabwe, Mugabe et al. (2010) showed that the accuracy of using indigenous knowledge depends on the ability of households to interpret the signs correctly. However, the elderly in the community are the ones who mainly possess local indigenous knowledge (Peters 2015). Indigenous knowledge is passed on from generation to generation, and the modernisation of rural areas is not supportive of this exchange of knowledge (Theodory 2014). Indeed, in this study, some study participants indicated there was limited passing on of indigenous knowledge to youths, as the youths were not interested. Theodory (2014) further supports this and indicates that indigenous knowledge is under threat of disappearance because of the increasing passing on of the elderly population who are crucial custodians of it.

Preference by households for scientific sources might be a product of the perceived reliability or accessibility of seasonal weather forecast and drought prediction. Preference for both sources could be an appreciation of the fact that either of the sources has advantages and shortcomings; hence, combined use enhances complementarity. Though scientific weather information is important, indigenous knowledge is recognised as providing economical and effective solutions that are likely sustainable, as well as bearing ethical and heritage values (Janicot et al. 2015).

## Conclusion

An important conclusion drawn from this article is that weather and drought prediction information disseminated through radio and newspapers was not sufficiently simplified to be understood by ordinary smallholder farmers. In addition, some households do not trust seasonal weather forecasts disseminated through media, as they have been proved to be inaccurate in the past. The lack of trust on weather information might result in non-action. More so translation of information into action is somewhat limited as smallholder farmers face difficulties in making informed decisions for livelihood activities and lack the means to adapt accordingly. FAO (2014) notes that early warning information only reduces the impact of a drought on households’ livelihood activities if appropriate action is taken. Furthermore, some households were not receiving seasonal weather forecasts timely for them to assess the implication of the forecasts, and make informed decisions to protect livelihood strategies and assets. This has in many cases left households uncertain on what to prepare for, creating a platform for drought disaster occurrence. The key to reducing risk is having timely access to weather information on the nature of the impending hazard and being prepared for it, and this might be the missing ingredient among many rural smallholder farmers (Simba et al. 2012; Twigg 2015).

The use of indigenous knowledge was based on the ability to read the signs and decode the information for consideration by smallholder farmers. Theodory (2014) argues that if value is put on indigenous knowledge by government and development organisations and used effectively, households will be in a position to make their own seasonal weather forecast and prediction without wasting time waiting for scientific weather forecasts. Perhaps more widespread appreciation of this activity would encourage and empower local communities to preserve this knowledge even more especially as correct interpretation of signs is an important aspect of household decision-making for farming in rural areas. Hiwasaki et al. (2014) maintain that the resilience of communities facing climate risks can increase when scientific and indigenous knowledge are combined. Therefore, there is need to document indigenous knowledge for weather forecasting and drought prediction to facilitate its application through official channels (Mercer et al. 2009). Acknowledging the existence of indigenous knowledge is within the fundamentals of Hyogo Framework for Action 2005–2015, which views ‘traditional and indigenous knowledge and cultural heritage’ as that of ‘knowledge innovation and education needed to build a culture of safety and resilience at all levels’. In conclusion, Theodory (2014) argues that indigenous knowledge is valuable to climate science as it enhances observations and interpretations on a larger spatial scale with considerable temporal depth by highlighting elements that are measured by climate science.

## Footnotes

1 The respondents for this study were the adult of each of the households, either the household head or the spouse. Considering the sampling approach was such that the households with no respondents were replaced with the nearest households, hence reaching the targeted sample size.

2 Indigenous knowledge for this study is defined as local knowledge generated over a period of time through people’s close interaction with nature. Thus, it is indigenous to a selected geographical location and its inhabitants (Theodory 2014).
